# Discrepancies between Vaccine Documentation and Serologic Status for Diphtheria, Tetanus, and Hepatitis B in Internationally Adopted Children

**DOI:** 10.3390/vaccines8030489

**Published:** 2020-08-30

**Authors:** Angela Bechini, Sara Boccalini, Ilaria Rancan, Luisa Galli, Beatrice Zanella, Elena Chiappini

**Affiliations:** 1 Department of Health Sciences, University of Florence, 50134 Florence, Italy; angela.bechini@unifi.it (A.B.); sara.boccalini@unifi.it (S.B.); beatrice.zanella@unifi.it (B.Z.); 2 Meyer Children’s University Hospital, Department of Health Sciences, University of Florence, 50139 Florence, Italy; ilaria.rancan@stud.unifi.it (I.R.); luisa.galli@unifi.it (L.G.)

**Keywords:** seroprevalence, immunological status, vaccine, internationally adopted children, hepatitis B, tetanus, diphtheria

## Abstract

Internationally Adopted Children (IAC) often show suboptimal immunisation coverage, but available data are discordant. Data at the first evaluation of 2073 IAC (median age: 6 years) referred to the Meyer Children’s University Hospital (Florence, Italy) in 2009–2019 were analysed in order to evaluate their immunisation status against diphtheria, tetanus, and hepatitis B. Negative antibody titres were observed in 11.5% of the IAC for diphtheria, 18.6% for tetanus, and 39.0% for hepatitis B. At multivariate analysis, originating from Africa was an independent risk factor for seronegativity for the three diseases (*p* < 0.001), while age below four years was an independent factor associated with protective immunity, only considering hepatitis B (*p* < 0.001). Vaccine documentation was an additional factor independently associated with protective immunity. However, a discrepancy between documentation (indicating previous vaccinations) and serology (showing negative antibody titres) was evidenced in 3.8% of the children for diphtheria, 12.6% for tetanus, and 29.6% for hepatitis B. This finding suggests that although vaccine documentation may reflect the presence of protective antibody titres, it should not be accepted as absolute evidence of protective immunity, underlining the importance of a complete assessment of immunisation status in IAC, particularly in those originating from Africa and aged over four years.

## 1. Introduction

The appropriate immunisation against vaccine-preventable diseases (VPDs) is a priority for healthcare systems. In 2018, global coverage with the recommended three doses of diphtheria-tetanus–pertussis (DTP) vaccine was estimated at 86% [1], while global coverage with three doses of hepatitis B (HBV) vaccine was at 84% [2]. In Italy, 2018 coverage rates exceeded 95% for tetanus (95.5%), diphtheria (95.4%), and hepatitis B (95.2%) vaccines, reaching the 95% coverage target set in the National Immunisation Plan [3].

Internationally Adopted Children (IAC) often present suboptimal immunisation against VPDs [4,5]; some of them did not receive any vaccine or were only partially vaccinated. Additionally, documentation of their immune status is not always available [6], and even when vaccination status is known, documentation may not correspond with the presence of protective antibody titres. The lack of vaccine documentation reliability has been reported in the literature, and numerous studies correlate this unreliability with no protection or under-immunisation [5,7,8,9].

Italy is the second nation at a global level with the highest rate of international adoptions behind the United States of America; in 2019, 1205 children were internationally adopted in Italy [10].

As regards diphtheria and tetanus immunisation, the Italian protocol recommends considering vaccine documentation as valid to assess tetanus immunisation status if six or more doses of DTP are reported in the documentation. A serological test should be performed if vaccine documentation is unavailable or unreliable for children younger than seven years of age. Antibody titre more than or equal to 0.1 IU/mL is considered protective, and this is also suggestive of immunity against diphtheria [11]. Additionally, the Spanish protocol recommends relying on the documentation and performing serology only in selected cases [12], while the French protocol suggests performing a tetanus antibody assay four to eight weeks after a dose of tetanus vaccine [6]. In the American Academy of Pediatrics (AAP) protocol, serology tests for diphtheria and tetanus are requested for IAC under six months of age with or without written documentation of immunisation [13].

Relying on previous documentation is not always useful; in the literature, concordance between serology tests and documentation of the vaccines was reported at 61–95% for tetanus, and 70–98% for diphtheria [8,9,14,15,16,17].

Tetanus protective antibody levels were observed in 35–97% and for diphtheria in 35–93% of the IAC [8,9,14,15,16,17,18,19,20,21,22,23].

Hepatitis B protection rate among IAC reported in the literature was extremely variable at 12–89% [5,8,14,15,16,17,18,19,20,21,24,25,26,27,28]. Additionally, the prevalence of HBV infection among IAC has been estimated to be around 2% to 5% [26].

In Italy, similarly to other countries, a complete assessment of HBV serology is recommended for IAC. Some differences can be highlighted: while Italy and Canada protocols suggest performing the research for HBsAg (hepatitis B surface antigen) and HBsAb (anti-hepatitis B surface antibody), United Kingdom, Ireland, France, Spain, US AAP and CDC (Centers for Disease Control and Prevention), and ECDC (European Centre for Disease Prevention and Control) suggest to research also HBcAb (anti-hepatitis B core antibody) [4,6,7,12,13,29,30,31]. Testing for HBcAb makes the HBV-assessment more complete. Positive HBcAb and negative HBsAg can be detected in four different conditions: acute HBV infection, low levels of HBsAb in the serum that are not detectable by serotesting, susceptibility to HBV because of a false positive HBcAb, and undetectable level of HBsAg present in serum when the child is chronically infected [32].

Meyer Children’s University Hospital offers complete screening to all adoptees at their arrival in Italy, including serology for measles, mumps, rubella, varicella, tetanus, diphtheria, and hepatitis B. Serology for other VPDs is not routinely performed, because testing antibody titres is not possible for every disease [29]. As regards pertussis, antibody titres do not correlate with immune status, and the utility of testing anti-*Bordetella pertussis* serum antibody titres to document infection as well as response to immunization is under debate [33]. Therefore serology is not performed at our centre, and protective levels against diphtheria and tetanus are considered surrogate markers for pertussis immunity because these vaccines are usually combined [29]. *Rotavirus*, pneumococcal, and meningococcal vaccines are recommended for all IAC since it is not possible to evaluate their antibody titres [18], and these vaccines are often not available or given in the birth countries of IAC [29]. The same strategy is applied for type B *Haemophilus influenzae* vaccine in children who are less than 5 years of age. For immunocompetent children >5 years old, no dose is indicated, except for certain high-risk older children who may still require vaccination [13]. As regards *poliovirus* infection, a neutralizing antibody test (NT) for *poliovirus* types 1, 2, and 3 could confirm immunity to *poliovirus* [13], but NT requires the use of tissue cultures and live viruses. Thus, its use is problematic [34], and it is not routinely performed in our centre.

We already published a study on the immunization status of IAC against measles, mumps, rubella, and varicella and documented a discrepancy between serology and vaccine documentation attesting previous vaccination in more than a quarter of IAC [35]. In this study, we analysed the available vaccination documentation, and the immunological status for diphtheria, tetanus, and hepatitis B in a large population of IAC referred to a single centre in Tuscany (Italy) over 11 years.

## 2. Materials and Methods

### 2.1. Study Population

Between January 2009 and December 2019, we enrolled all the IAC consecutively referred to the Anna Meyer Children’s University Hospital by their adoptive parents, who consented to the screening evaluation. These children were assessed following the protocol for the first screening recommended by the GLNBI (National Working Group for Immigrant Children), as previously described [14], in particular focusing on immunisation status.

Italian adopted children were excluded from the study. Conversely, IAC from all over the world who were under 18 years of age and who had at least one serological test among hepatitis B, diphtheria, and tetanus performed at the first evaluation were included.

The study received approval from the Meyer Children’s University Hospital Ethics Committee; the Ethical Code Number is 15/2010. Parents who consented to their adoptive children participating in the study signed written informed consent.

### 2.2. Study Design

This is a retrospective monocentric study. For each child included in the study, the following information was collected and entered into an electronic database, following a standard operative protocol for IAC developed internationally and adopted at Meyer Children’s University Hospital [13,14,20]. The electronic database is a secure system to protect patient information. Briefly, the following information was retrieved for the present study: country of origin, gender, age at first observation, vaccine documentation, and results of serological tests for diphtheria, tetanus, and hepatitis B. At the first evaluation, all the children underwent a venepuncture and laboratory assessment including serologic tests. Staff involved in the venepuncture and laboratory assessment was appropriately trained to do so.

All the other laboratory examinations were performed in the same laboratory at the Meyer Children’s University Hospital using standardised techniques and according to manufacturers’ instructions.

In particular, serology tests for diphtheria and tetanus were performed using enzyme-linked immunosorbent assay (ELISA) technology (ETI-Max 3000, DiaSorin, Saluggia (V.C.), Italy) with a definition of seropositive samples when antibody titres were >0.1 IU/mL. Serology tests for hepatitis B were performed using chemiluminescent immunoassay (CLIA) technology (LIAISON XL Anti-Hbs II System, DiaSorin, Saluggia (V.C.), Italy) with a definition of the seropositive sample when antibody titres were >10 mIU/mL.

### 2.3. Seroprevalence of Antibody Protection against Diphtheria, Tetanus and Hepatitis B

Children were subdivided according to age into five groups (<1 year, 1–4 years, 5–9 years, 10–14 years, 15–18 years) and classified as seropositive or seronegative against each specific VPD based on serotesting results. Antibody seroprevalence for each age group was calculated and expressed as a percentage.

### 2.4. Concordance between Vaccine Documentation and Serotesting Results

Vaccine documentation was assessed considering the number of doses recorded and the concordance between the available documentation and serotesting results for diphtheria, tetanus, and hepatitis B. These data were recorded and entered in tables.

### 2.5. Statistical Analysis

Data were reported as the median and interquartile range (IQR) or absolute numbers and percentages. Fisher exact test and Chi-square test were used to compare categorical variables, as appropriate.

Univariate and multivariate logistic regression analyses were performed to evaluate the association between the absence of protective antibody titres and possible risk factors. Factors significantly associated with seronegativity at univariate analysis (*p* < 0.05) were included in the multivariate analysis.

All statistical analyses were carried out using the SPSS (Statistical Package of Social Sciences, Chicago, IL, USA) for Windows software program version 19.0. A *p*-value <0.05 was considered significant.

## 3. Results

### 3.1. Characteristics of the Study Population

In the period 2009–2019, 2299 IAC from 68 countries were assessed for post-adoption screening. Of this initial group, 2073 were eligible for our study, while 226 were excluded from the evaluation because at least one serological test result was not available.

Considering serology results and documentation available, 174 IAC were assessed for diphtheria, 2033 for tetanus, and 2052 for hepatitis B. Thus, 8.4% and 98.1% of the study population were included in the study for diphtheria and tetanus, respectively, while for hepatitis B, children included in the analysis represented 99% of the study population. The median age at first evaluation was 5.99 (IQR: 3.33–8.21) years, and 40.7% of the children were girls (843/2073) (Table 1).

Forty percent of the IAC were adopted from Europe (844/2073), and most of them were from Russia; 21.7% were from Central or South America (449/2073); 20.3% were adopted from Asia (420/2073); 17.4% were from Africa (360/2073) (Table 1). The most represented countries were Russia (477/2073, 23.0%), Colombia (158/2073, 7.6%), India (157/2073, 7.6%), and Ethiopia (132/2073, 6.4%). Since the most populated area of Russia is in Europe (and not in Asia), the data from Russia were entered in Europe. Younger children came from Asia, while the older IAC were born in America (Table 1).

Children aged one year or younger accounted for 2.8% of IAC, children between one and four years accounted for 39.0% of IAC, and children aged between 5 and 9 years accounted for 47.5% of IAC. Only 9.3% of the IAC were 10 to 14 years old, and older IAC belonging to the age group 15–18 years accounted for 1.4% of the population. IAC aged between five and nine years represented the largest age group (Table 2).

### 3.2. Evaluation of the Immunological Coverage Using Serological Tests

Most of the children enrolled by Meyer Children’s University Hospital during the study period (2009–2019) were tested to evaluate the protective antibodies titres against diphtheria, tetanus, and hepatitis B. Protective antibody titres were recorded in 88.5% (154/174) of IAC for diphtheria, in 81.4% (1655/2033) for tetanus, and in 61.0% (1252/2052) for hepatitis B. Moreover, Africa was the continent with the highest percentage of seronegative children for diphtheria (*p* < 0.001), tetanus (*p* < 0.001), and hepatitis B (*p* < 0.001) (Table 3).

**Figure 1:**
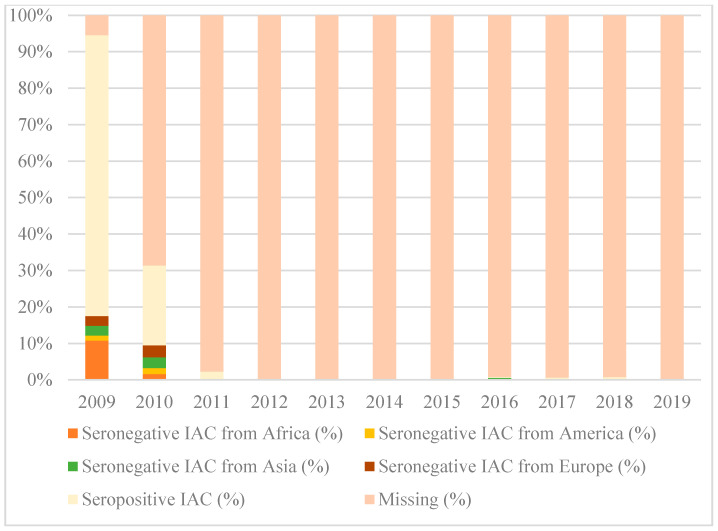
Percentages of seropositive and seronegative children in the study for diphtheria, by year of the first visit in Italy and country of origin.

**Figure 2:**
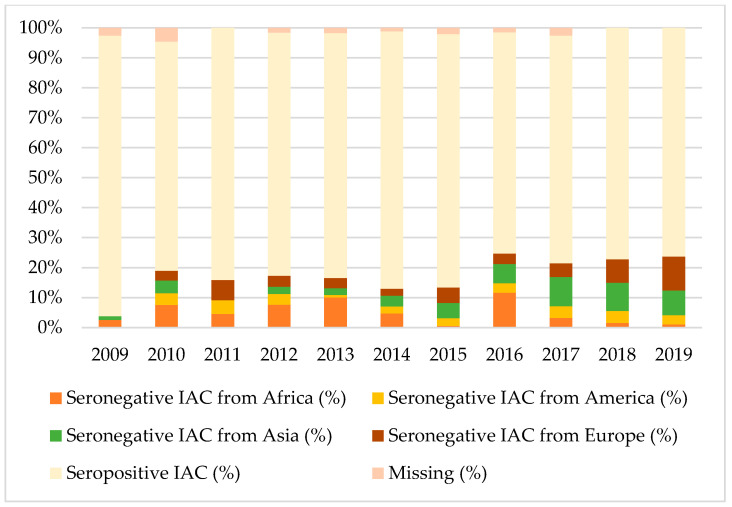
Percentages of seropositive and seronegative children in the study for tetanus, by year of the first visit in Italy and country of origin.

**Figure 3:**
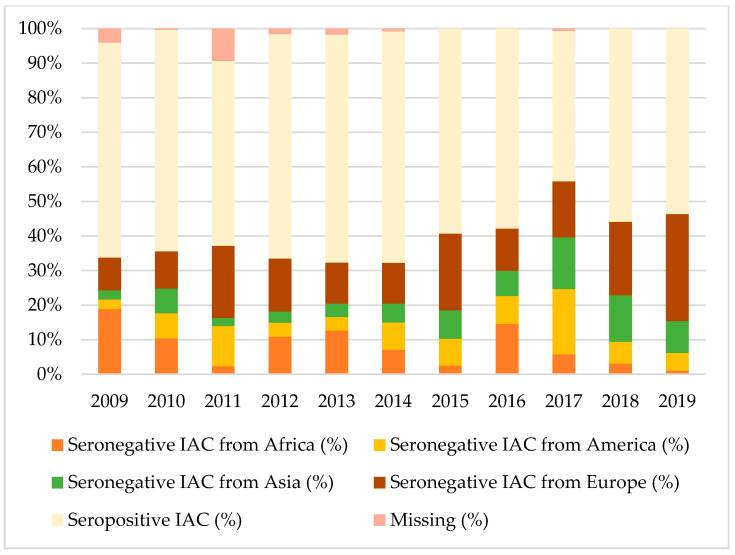
Percentages of seropositive and seronegative children in the study for hepatitis B, by year of the first visit in Italy and country of origin.

Figure 1, Figure 2 and Figure 3 show the percentages of seropositive and seronegative IAC by year of the first visit in Italy when serology was performed. Seronegative IAC were additionally divided by country of origin.

As regards to diphtheria, serology was performed in most IAC only in 2009, when the majority of seronegative IAC were from Africa (10.8% of all IAC arrived in 2009).

Considering tetanus and hepatitis B, most seronegative IAC were from Africa, Europe, or Asia, depending on the year of the first visit, except for 2017 when American IAC represented the majority of seronegative IAC for hepatitis B.

Table 4 shows the percentages of seropositive IAC for tetanus, diphtheria, and hepatitis B among those with serology performed, compared to vaccination coverage in 2019 [36,37], by country of origin. Since IAC in our study came from 68 different countries, we considered the 20 most represented countries of origin, including about 90% of the IAC in our study (1858/2073).

Data for diphtheria were difficult to interpret because serology was not performed for most IAC.

As regards tetanus, percentages of seropositive IAC for tetanus were higher than vaccination coverage only for IAC originating from Colombia (96% of seropositive IAC, while vaccination coverage with the third dose of DTP vaccine in 2019 was 94%), and from Ukraine (87% of seropositive, while vaccination coverage with the third dose of DTP vaccine in 2019 was 80%). Seropositive IAC from Ethiopia, Congo, China, and Romania did not reach 60%.

As regards hepatitis B, percentages of seropositive IAC were always lower than vaccination coverage in the different countries with the third dose of hepatitis B vaccine. Seropositive IAC from Ethiopia, Congo, Hungary, and Romania did not reach 40%.

**Figure 4:**
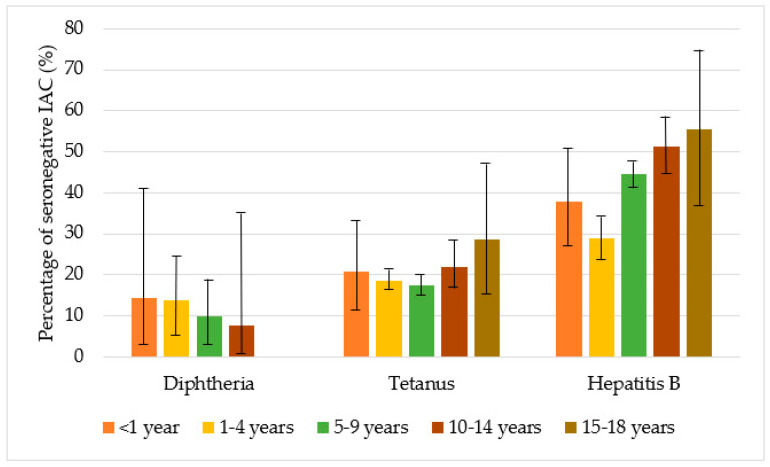
Percentage of seronegative children in the study with for diphtheria, tetanus, and hepatitis B, by age group, with 95% CI (confidence interval) (Agresti–Coull method).

Figure 4 shows the percentages of seronegative children in the study for diphtheria, tetanus, and hepatitis B, by age group. Protection against diphtheria ranged from 85.7% to 100% and protection against tetanus from 71.4% to 82.5%, with no statistically significant differences in the different age groups (*p* > 0.05). Protection against hepatitis B was higher for children aged from one to four years (71.2%) than for children of the other age groups (*p* < 0.001), and in general, IAC up to nine years were more protected against hepatitis B than older children (*p* < 0.001) (Table A1 in the Appendix A).

Documentation of the vaccine received in the country of origin was not always available. In particular, a discrepancy between documentation indicating previous vaccinations and serology showing non-protective antibody titres was evidenced in 3.8% of the children for diphtheria, 12.6% for tetanus, and 29.6% for hepatitis B (Table 5). 

**Figure 5:**
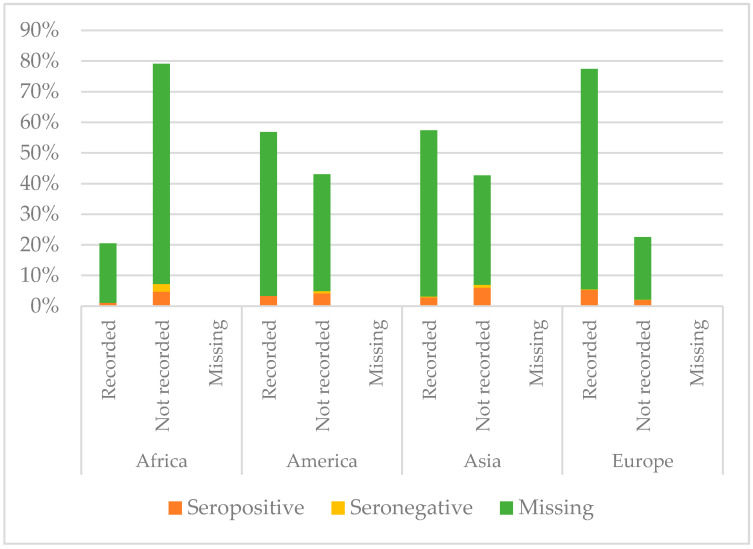
Comparison between immunisation status of IAC reported in the documentation and serology for diphtheria, by continent of origin (Table A2 in the Appendix A).

**Figure 6:**
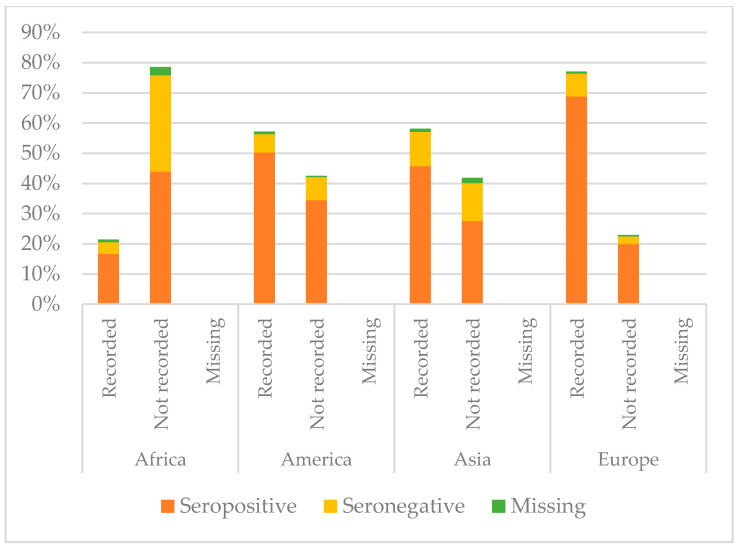
Comparison between immunisation status of IAC reported in the documentation and serology for tetanus, by continent of origin (Table A3 in the Appendix A).

**Figure 7:**
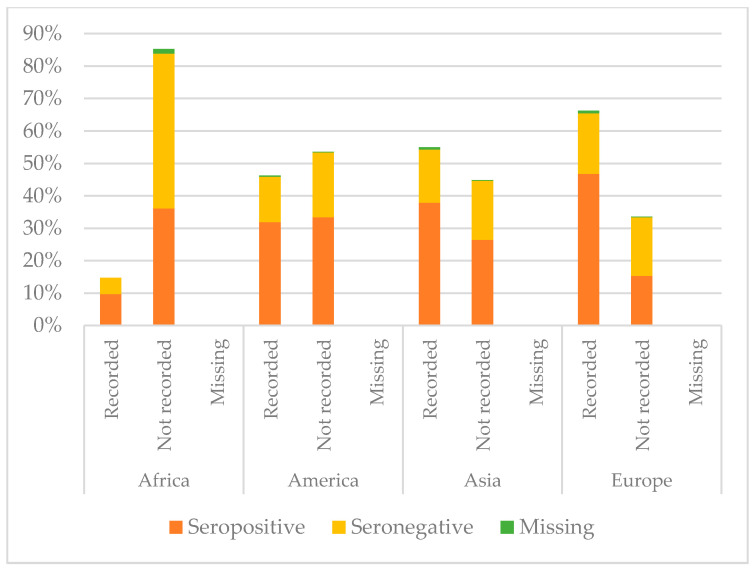
Comparison between immunisation status of IAC reported in the documentation and serology for hepatitis B, by continent of origin (Table A4 in the Appendix A).

Figure 5, Figure 6 and Figure 7 show the comparison between the immunisation status of IAC reported in the documentation and serology, by continent of origin.

Serology for diphtheria was not performed for most of the IAC considered in this study; only 174/2073 had available serology results. Among those with serology tested, European children showed higher protection than the others (*p* = 0.012): 95.4% of European children with serology performed were seropositive. On the contrary, IAC with lower protection rates were those from Africa (*p* < 0.001); 30% of African IAC with serology performed were seronegative. They also were the group with less documentation of vaccination against diphtheria (*p* < 0.001); 79.4% of African children did not have documentation. However, children who presented with documented previous vaccination were more likely to be protected than those with no documentation (*p* = 0.004).

As regards tetanus, African IAC were less protected (60.56%, *p* < 0.001), and only 21.39% had the documentation of previous vaccination. Instead, coming from Europe correlated with higher protection (*p* < 0.001); 88.8% of the children coming from Europe presented with protective antibody titres. Moreover, 19.9% of the European IAC were protected, even if they did not have a recorded vaccination, and actually for them having documentation did not correlate with the major probability to be protected. Conversely, children coming from Africa, Asia, and America were more likely to be protected against tetanus if they had the documentation of a previous vaccination (*p* < 0.05). However, among those with recorded vaccination, there were some children without protective antibody titres: 3.9% for Africa, 6.0% for America, 11.4% for Asia, and 7.6% for Europe.

Considering hepatitis B, IAC from America presented higher protection against hepatitis B (65.3%) than the others (*p* = 0.022), while coming from Africa correlated with lower protection (*p* < 0.001) and a higher probability to have no documentation of hepatitis B vaccination if compared with children coming from the other continents (*p* < 0.001). Indeed, 52.8% of the IAC originating from Africa were seronegative and 85.3% did not present any documentation. Among children with recorded vaccination, some had no protective antibody titres: 5% of the children from Africa, 14.0% of those from America, 11.3% of those coming from Asia, and 18.7% of the IAC from Europe. However, protection for children with recorded vaccination for hepatitis B was higher than for those without it, except for children coming from America (*p* = 0.125).

**Figure 8:**
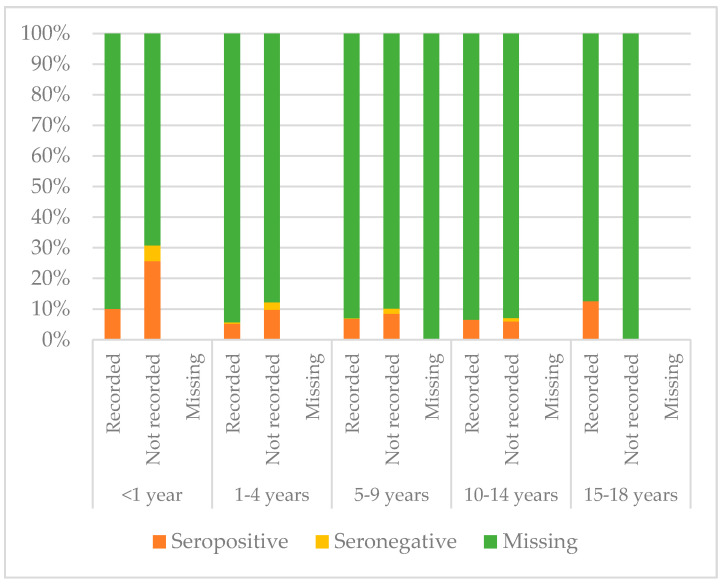
Comparison between immunisation status of IAC reported in the documentation and serology for diphtheria, by age group (Table A5 in the Appendix A).

**Figure 9:**
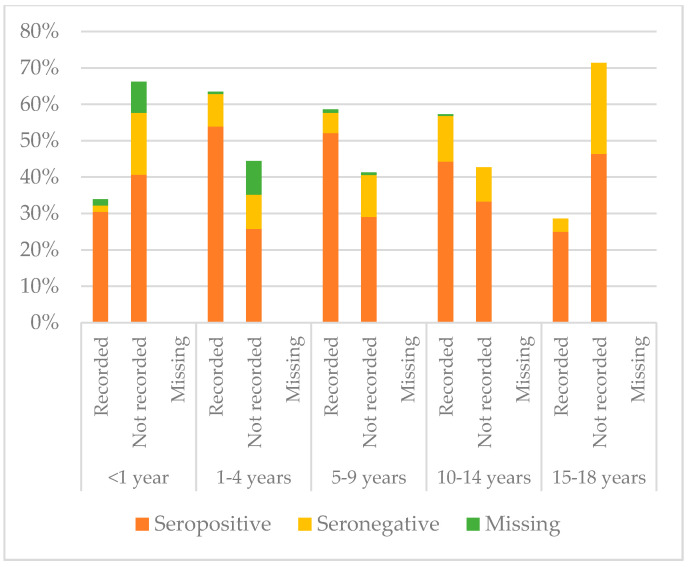
Comparison between immunisation status of IAC reported in the documentation and serology for tetanus, by age group (Table A6 in the Appendix A).

**Figure 10:**
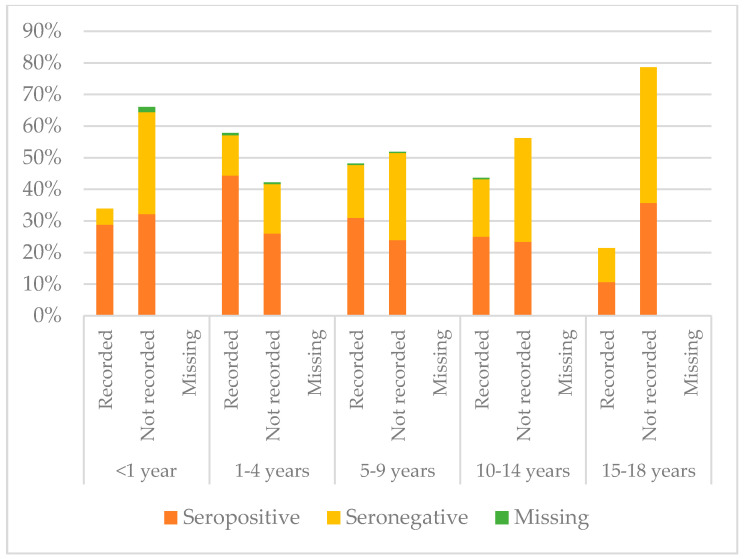
Comparison between immunisation status of IAC reported in the documentation and serology for hepatitis B, by age group (Table A7 in the Appendix A).

Figure 8, Figure 9 and Figure 10 compare immunisation status of IAC reported in the documentation to serology, dividing IAC by age groups. The highest proportion of children with documentation was in the group of children aged 1–4 years; 63.3% of them had a recorded vaccination against diphtheria (*p* = 0.002), 63.53% against tetanus (*p* = 0.002), and 57.9% against hepatitis B (*p* < 0.001). On the other hand, IAC aged 15–18 were the group with less documentation; only 28.6% for diphtheria and tetanus (*p* = 0.001) and 21.4% for hepatitis B (*p* = 0.003) provided documentation.

IAC without documentation of previous vaccinations were 41.0% (849/2073) for diphtheria, 40.7% for tetanus (844/2073), and 49.1% for hepatitis B (1018/2073). Moreover, 66.1% of IAC up to one year of age (39/59) had no documentation of vaccination for diphtheria, tetanus, and hepatitis B, while considering IAC older than 14 years, no documentation was available for 71.4% (20/28) of the adoptees, considering tetanus and diphtheria, and for 67.9% (19/28) of the IAC for hepatitis B. Among IAC with documented doses, the majority received four doses of diphtheria and tetanus vaccines, while as regards hepatitis B, most IAC received three doses (Table 6).

### 3.3. Analysis of Seronegativity

Univariate and multivariate logistic regression analyses were performed in order to evaluate the association between seronegativity for tetanus, diphtheria, and hepatitis B and possible risk factors (Table A8, Table A9 and Table A10 in the Appendix A).

As regards to tetanus, logistic regression analysis included 2033 IAC.

Factors significantly associated with seronegativity were origin from Asia vs. origin from Europe (OR: 2.86; 95% CI: 2.08–3.92; *p* < 0.001), and origin from Africa vs. origin from Europe (OR: 5.22; 95% CI: 3.82–7.12; *p* < 0.001). Having documentation of previous vaccinations was significantly associated with the presence of protective antibody titres (OR: 0.38; 95% CI: 0.31–0.48; *p* <0.001). Arrival in Italy before 2009 vs. arrival in Italy in the period 2009–2011 was also significantly associated with the presence of protective antibody titres (OR: 0.19; 95% CI: 0.04–0.79; *p* = 0.022).

At multivariate logistic regression analysis, independent factors significantly associated with seronegativity were origin from Asia vs. origin from Europe, (aOR: 2.42; 95% CI: 1.75–3.35; *p* < 0.001), origin from Africa vs. origin from Europe (aOR: 3.61; 95% CI: 2.57–5.07; *p* < 0.001), and arrival in Italy in the period 2015–2019 vs. arrival in Italy in the period 2009–2011 (aOR: 1.43; 95% CI: 1.04–1.96; *p* = 0.028). Having documentation of previous vaccinations was an independent factor significantly associated with the presence of protective antibody titres (aOR: 0.50; 95% CI: 0.39–0.65; *p* < 0.001). Arrival in Italy before 2009 vs. arrival in Italy in the period 2009–2011 was also an independent factor associated with the presence of protective antibody titres (aOR: 0.18; 95% CI: 0.04–0.75; *p* = 0.019).

As regards to diphtheria, logistic regression analysis included 149 IAC. Of the group assessed in our study, 25/174 were not included in the logistic regression analysis, because they had borderline antibody titres against diphtheria (0.01–0.09 IU/mL).

Origin from Africa vs. origin from Europe was significantly associated with seronegativity (OR: 9.94; 95% CI: 2.40–41.16; *p* = 0.002). Having documentation of previous vaccinations was significantly associated with the presence of protective antibody titres (OR: 0.316; 95% CI: 0.10–1.00; *p* = 0.049).

At multivariate logistic regression analysis, an independent risk factor significantly associated with seronegativity was origin from Africa vs. origin from Europe (aOR: 5.55; 95% CI: 1.23–25.05; *p* = 0.026).

As regards to hepatitis B, logistic regression analysis included 2052 IAC.

Factors significantly associated with seronegativity were age 5–9 vs. age 0–4 (OR: 1.93; 95% CI: 1.59–2.34; *p* < 0.001), age 10–18 vs. age 0–4 (OR: 2.56; 95% CI: 1.89–3.47; *p* < 0.001), origin from Africa vs. origin from Europe (OR: 1.94; 95% CI: 1.51–2.49; *p* < 0.001), and arrival in Italy in the period 2015–2019 vs. arrival in Italy in the period 2009–2011 (OR: 1.32; 95% CI: 1.04–1.67; *p* = 0.024). Having documentation of previous vaccinations was significantly associated with the presence of protective antibody titres (OR: 0.44; 95% CI: 0.37–0.53; *p* < 0.001).

At multivariate logistic regression analysis, independent factors significantly associated with seronegativity were age 5–9 vs. age 0–4 (aOR: 2.09; 95% CI: 1.69–2.57; *p* < 0.001), age 10–18 vs. age 0–4 (aOR: 2.69; 95% CI: 1.94–3.72; *p* < 0.001), origin from Africa vs. origin from Europe (aOR: 1.60; 95% CI: 1.20–2.13; *p* = 0.001), and arrival in Italy in the period 2015–2019 vs. arrival in the period 2009–2011 (aOR: 1.44; 95% CI: 1.12–1.86; *p* = 0.005). Independent factors significantly associated with the presence of protective antibody titres were origin from America vs. origin from Europe (aOR: 0.63; 95% CI: 0.48–0.81; *p* < 0.001), having documentation of previous vaccinations (aOR: 0.49; 95% CI: 0.40–0.59; *p* < 0.001), and arrival in Italy before 2009 vs. arrival in Italy in the period 2009–2011 (aOR: 0.50; 95% CI: 0.25–0.99; *p* = 0.046).

## 4. Discussion

In the present study, we analysed the vaccination documentation and serological status for diphtheria, tetanus, and hepatitis B in a large population of more than 2000 IAC referred to a single centre in Tuscany (Italy), over 11 years. Seronegative children were 11.5% for diphtheria, 18.6% for tetanus, and 39% for hepatitis B. These figures were more pronounced considering African children; 30% of them were seronegative for diphtheria, 37.5% for tetanus, and 53.5% for hepatitis B. Originating from Africa was an independent factor significantly associated with seronegativity for all three diseases. IAC originating from Africa were also the children having less frequent documentation of previous vaccinations. On the contrary, the higher percentage of protected children was among European IAC for diphtheria and tetanus and among those originating from America for hepatitis B.

In a previous Italian multicentre study involving about 700 African IAC, non-protective anti-tetanus and anti-hepatitis B antibody titres were observed in 35% and 52% of children [20]. However, the reason for such a finding was not clear. Most African IAC included in our study originated from Ethiopia (132/360) and the Democratic Republic of the Congo (90/360), and seronegative IAC from these two countries were more than 40% for tetanus and more than 60% for hepatitis B. Data for diphtheria were insufficient to allow appropriate evaluation of serologic immunity, by country of origin, but tetanus serology could be also considered suggestive of the immunity against diphtheria [11]. Interestingly, Van Kesteren et al. reported that most Ethiopian children were not adequately immunised—only 34% of the adoptees were protected for hepatitis B [27]—while in a study by Miller et al., only 12% of Ethiopian children displayed protective anti-HBV antibody titres [23]. In our study, the highest proportion of seronegative children against HBV from Africa was retrieved in 2009, and most of the adoptees were actually from Ethiopia (14/20). The highest proportion of children coming from the Democratic Republic of the Congo was in 2016, after the removal of the ban for international adoption established in 2013. In 2016, a large proportion of seronegative children for tetanus and hepatitis B were from Africa, and in that year children from the Democratic Republic of the Congo in our dataset were about 39% of all African IAC. We speculated that the high rate of seronegative IAC might be influenced by the period of political instability in the country.

Considering the different age classes, there was no significant difference as regards to protection against diphtheria and tetanus, while children aged 0–4 years were the most frequently protected for hepatitis B; age 5–9 and age 10–18 were independent factors significantly associated with seronegativity for HBV. Interestingly, according to their documentation, most IAC aged <1 year received no dose of vaccine. Similar results were found in a recent Italian study in which the seroprevalence against hepatitis B was assessed in sera samples collected at Meyer Children’s University Hospital from the paediatric and adolescent population (1–18 years) of the Province of Florence. Indeed, children under six years of age presented the highest level of anti-HBs (80% of seropositivity in the age group) [38].

Several previous studies reported the proportion of IAC with protective antibody titres against different VPDs. In particular, observed protection rates ranged between 35% and 93% for diphtheria, 35% and 97% for tetanus, 12% and 89% for hepatitis B [5,8,9,14,15,16,17,18,19,20,21,22,23,24,25,26,27,28]. This variability may likely be attributed to the heterogeneity of the study populations, due to the differences both in the number of children included in the studies and in the countries of origin of the IAC enrolled. In particular, the study by Staat et al. including about 700 children originating almost from all continents was one of the most similar to our study among those considered, as regards to the characteristics of the study population. Interestingly, in this study, the protection rates for diphtheria, tetanus, and hepatitis B were not far from our data: 80% for diphtheria, 89% for tetanus, and 60% for hepatitis B [17]. The same seroprevalence rate for hepatitis B was also found in Italian children by Zanella et al. [38].

Written documentation of previous vaccination not always reflected in immunisation status of IAC; seronegative children among those with documented vaccination were about 4% for diphtheria, 13% for tetanus, and reaching almost 30% for HBV. These results were in accordance with data reported in the literature. Previous studies described a discrepancy between documentation (recorded vaccination) and serology (negative antibody titres) in 2–30% of IAC for diphtheria, 5–39% of IAC for tetanus, and 6–55% for hepatitis B [5,8,9,14,15,16,17,18,19,24,28]. In particular, concerning hepatitis B vaccination, this discrepancy may be explained by the fact that time since the last dose of vaccine received is predictive of the level of anti-HBs titres. Children who had the anti-hepatitis B vaccination 6–10 years before tend to have lower antibody levels than those who had received the last dose of the hepatitis B vaccine in the previous five years. Thus, the more recent the vaccination, the more likely is a seropositive result for anti-HBs [38].

Documentation could be incomplete [13] or not easy to understand because of the different language or wrong translations. It could be also false, in the case of vaccines reported to be administered before the IAC date of birth, or when dates of vaccine administration are written with the same handwriting and ink, or on the same day of subsequent months [6,12]. In addition, even when documentation results are trustworthy, vaccination may be not effective due to improper vaccine storage or a child’s impaired immunological status, secondary to malnutrition or infections [35].

The predictive value of the immunisation records of IAC was investigated in several studies; some of them have shown that immunisation records may not reflect the actual protective immunity [5,14,19], while others have reported that having documentation was generally correlated with higher probability to be protected [16]. In our study, univariate and multivariate logistic regression analyses showed that having vaccine documentation was an independent factor significantly associated with protective immunity.

Unreliability of vaccination records must be considered when deciding the immunisation approach to guarantee IAC protection against VPDs. At present, numerous strategies for their immunisation have been suggested with no clear conclusions [14,20].

Moreover, pre-vaccine serology has a high risk of underestimating actual vaccine coverage; antibody levels can decrease over time without losing memory cells. For this reason, some authors suggested performing a single-dose administration of vaccines in order to generate a booster-type response in most migrant children. Fougère and colleagues, in a recent article including 208 immigrant children, found that one booster dose was sufficient to obtain protective antibody serum levels in 98% of children for tetanus [39]. Indeed French protocol suggests performing tetanus antibody assay 4 to 8 weeks after a dose of tetanus vaccine [6].

This issue could be particularly important when considering HBV vaccination. A multicentre study by Zanetti et al. showed that most individuals with antibody titres less than 10 IU/L had an anamnestic increase in the concentration of anti-Hbs after the administration of a booster dose [40]. A booster dose is not necessary to maintain protection in previously vaccinated individuals [41], but it can be used in order to detect immune children among those with low antibody titres, in particular seronegative IAC with documentation of HBV vaccine. For example, Spanish protocol suggests to directly administer one dose of HBV vaccine at the first clinical evaluation of IAC and thereafter to perform serology [12], while French protocol suggests performing a pre-vaccination serology test if the patient belongs to a population at risk of hepatitis B and thereafter performing an anti-HBs antibody assay four to eight weeks after a hepatitis B vaccine dose [6]. Indeed, this is the strategy used in our centre regarding the HBV vaccine, but not regarding other vaccines. However, this approach is a successful strategy recommended by the Italian National Immunization Plan only for some groups of workers at risk [42,43,44], while current national and international guidelines do not support this strategy in IAC [7,13]. 

Our study has some limitations. Most of the children referred to Meyer Children’s University Hospital included in the current study were evaluated as per protocol, but data were not always available for all the variables, because during the 11-year study period, there were some changes in the investigations included in the screening protocol, and some tests were not performed in the whole population. This was probably caused by an incomplete collection of data in medical records or by the physicians’ incomplete adherence to the screening protocol. In addition, it was not possible to recruit all IAC arriving in Tuscany, as some parents may have consulted their primary care paediatrician or no one at all. However, considering that 2941 children were internationally adopted in Tuscany in the period 2009–2019 [11,45,46], international adoptees enrolled in our study represent about 70% of all IAC in Tuscany over that period. Moreover, it must be considered that serology testing does not explore all the mechanisms on which immunological response is based, and having low antibody titres does not always correspond to non-protection. In particular, pre-vaccine serology could underestimate the real vaccine coverage It has been demonstrated that individuals who had lost their anti-HBs seropositivity still show immunologic T cell memory and that these T cells are able to trigger anti-HBs production of B cells activated by revaccination [47]. Lastly, it is not possible to differentiate the immune response derived from vaccination or recovery from natural infection.

## 5. Conclusions

In our study, more than eighty percent of the IAC showed protective antibody titres for diphtheria and tetanus, while about sixty percent were protected for hepatitis B. IAC with documentation were more likely to be protected than those without it. However, a discrepancy between documentation indicating previous vaccinations and serology showing negative antibody titres was evidenced in 3.8% of the children for diphtheria, 12.6% for tetanus, and 29.6% for hepatitis B. This finding suggests that although vaccine documentation may be correlated with the presence of protective antibody titres, it should not be considered as absolute evidence of protective immunity. Relying on the documentation without performing serology tests could not assure an appropriate immunisation of all IAC. This result underlines the importance of a complete assessment of immunisation status in IAC, after their arrival, to provide vaccination to all seronegative adoptees.

## Figures and Tables

**Table 1:** Characteristics of the study population by continent of origin median age, gender, and period of arrival in Italy.

Characteristics of the Study Population	Africa	Americas	Asia	Europe	Total
Population (*n*)	360 (17.4%)	449 (21.7%)	420 (20.3%)	844 (40.7%)	2073 (100%)
Median age (*y*)	4.59	7.49	4.52	5.89	5.85
Interquartile range (IQR)	2.63–6.75	5.28–9.31	1.99–7.10	3.81–8.30	3.34–8.20
Females (*n*)	154 (42.8%)	209 (46.5%)	192 (45.7%)	288 (34.1%)	843 (40.7%)
Males (*n*)	206 (57.2%)	240 (53.5%)	228 (54.3%)	556 (65.9%)	1230 (59.3%)
Period of arrival in Italy Before 2009 (*n*)	6 (1.67%)	8 (1.78%)	13 (3.10%)	23 (2.73%)	50 (2.41%)
2009–2011 (*n*)	76 (21.11%)	121 (26.95%)	90 (21.43%)	158 (18.72%)	445 (21.47%)
2012–2014 (*n*)	133 (36.94%)	137 (30.51%)	95 (22.62%)	337 (39.93%)	702 (33.86%)
2015–2019 (*n*)	124 (24.44%)	170 (37.86%)	207 (49.29%)	291 (34.48%)	792 (38.21%)
Year missing (*n*)	21 (5.83%)	13 (2.90%)	15 (3.57%)	35 (4.15%)	84 (4.05%)

**Table 2:** The continent of origin of the internationally adopted children (IAC) referred to Meyer Children’s University Hospital, Florence, in the period (2009–2019), by age group (%).

Age Group Year	Africa % (*n* = 360)	Americas % (*n* = 449)	Asia % (*n* = 420)	Europe % (*n* = 844)	Total % (*n* = 2073)
<1	(22) 6.1	(2) 0.5	(31) 7.4	(4) 0.5	(59) 2.8
1–4	(176) 48.9	(94) 20.9	(200) 47.6	(339) 40.2	(809) 39.0
5–9	(135) 37.5	(281) 62.6	(170) 40.5	(399) 47.3	(985) 47.5
10–14	(20) 5.6	(65) 14.5	(16) 3.8	(91) 10.8	(192) 9.3
15–18	(7) 1.9	(7) 1.6	(3) 0.7	(11) 1.3	(28) 1.4
Total	17.4%	21.7%	20.3%	40.7%	100

**Table 3:** Percentages of seronegative children in the study for diphtheria, tetanus, and hepatitis B by continent of origin.

Disease	Seronegative/Total Number of Children for Each Disease	Africa (*n* = 360)	Americas (*n* = 449)	Asia (*n* = 420)	Europe (*n* = 844)
Diphtheria *n/N* (%)	20/174 (11.5%)	9/30 (30%)	3/37 (8.1%)	5/42 (11.9%)	3/65 (4.6%)
Tetanus *n/N* (%)	378/2033 (18.6%)	130/347 (37.5%)	61/442 (13.8%)	101/409 (24.7%)	86/835 (10.3%)
Hepatitis B *n/N* (%)	800/2052 (39.0%)	190/355 (53.5%)	153/446 (34.3%)	146/416 (35.1%)	311/835 (37.2%)

**Table 4:** Seropositive IAC for tetanus, diphtheria, and hepatitis B among those with serology performed compared to vaccination coverage in 2019, by country of origin.

Country of Origin	IAC *n*	Seropositive/IAC with Serology Performed *n/N* (%)			Vaccination Coverage (2019) in the Country of Origin (%)	
		Tetanus	Diphtheria	Hepatitis B	DPT3 ^1^ [36]	HepB3 ^2^ [37]
Russia (Europe)	477	438/473 (92.6%)	22/29 (75.9%)	364/472 (77.1%)	97%	97%
Colombia (America)	158	148/154 (96.1%)	12/12 (100.0%)	109/156 (69.9%)	94%	94%
India (Asia)	157	129/154 (83.8%)	14/17 (82.4%)	104/155 (67.1%)	91%	91%
Ethiopia (Africa)	132	71/123 (57.7%)	7/19 (36.8%)	45/130 (34.6%)	96%	96%
Hungary (Europe)	117	102/117 (87.2%)	5/5 (100.0%)	8/117 (6.8%)	99% ^3^	− (99% ^3^ in 1999)
Congo (Africa)	90	46/88 (52.3%)	3/4 (75.0%)	32/89 (36.0%)	83%	83%
Brazil (America)	90	72/89 (80.9%)	12/17 (70.6%)	58/89 (65.2%)	73%	80%
Viet Nam (Asia)	90	62/84 (73.8%)	8/14 (57.1%)	55/89 (61.8%)	89%	89%
China (Asia)	68	39/68 (57.4%)	3/4 (75.0%)	45/68 (66.2%)	99%	99%
Chile (America)	66	56/65 (86.4%)	4/4 (100.0%)	35/66 (53.0%)	96%	96%
Peru (America)	66	48/66 (72.7%)	1/2 (50.0%)	49/66 (74.2%)	88%	88%
Ukraine (Europe)	65	54/62 (87.1%)	13/16 (81.3%)	39/63 (62.9%)	80%	77%
Burkina Faso (Africa)	54	44/54 (81.5%)	1/1 (100.0%)	34/52 (65.4%)	91%	91%
Bulgaria (Europe)	53	45/52 (86.5%)	5/6 (83.3%)	38/53 (71.7%)	− (92% in 2018)	− (85% in 2018)
Philippines (Asia)	44	37/43 (86.0%)	1/1 (100.0%)	26/43 (60.5%)	77%	77%
Poland (Europe)	44	40/44 (90.9%)	5/6 (83.3%)	33/43 (76.7%)	− (95% in 2018)	− (91% in 2018)
Lithuania (Europe)	29	24/29 (82.8%)	0/0	16/29 (55.2%)	92%	92%
Costa Rica (America)	24	21/24 (87.5%)	1/1 (100.0%)	15/24 (62.5%)	95%	− (98% in 2018)
Albania (Europe)	18	17/18 (94.4%)	0/0	9/18 (50.0%)	99%	99%
Romania (Europe)	16	8/16 (50.0%)	0/0	6/16 (37.5%)	88%	90%

(**^1^**) DPT3 = third dose of diphtheria toxoid, tetanus toxoid, and pertussis vaccine, (**^2^**) HepB3 = third dose of hepatitis B vaccine, (**^3^**) coverage was reported over 99.5%.

**Table 5:** Comparison between documentation recorded and serological tests performed in Italy.

Disease	Immunisation Status According to Documentation *n* (%)	Immunisation Status According to Serological Test *n* (%)		Total	*P*-Value
		Seropositive	Seronegative		
Diphtheria	Recorded	76 (96.2%)	3 (3.8%)	79	*p* = 0.004
	Not recorded	78 (82.1%)	17 (17.9%)	95	
Tetanus	Recorded	1059 (87.4%)	153 (12.6%)	1212	*p* < 0.001
	Not recorded	596 (72.7%)	224 (27.3%)	820	
Hepatitis B	Recorded	732 (70.4%)	308 (29.6%)	1040	*p* < 0.001
	Not recorded	518 (51.3%)	492 (48.7%)	1010	

**Table 6:** The number of documented doses for IAC, by age groups.

Disease	Age Group	Number of Doses								
		0 ^2^	1	2	3	4	5	6	7	Missing ^3^
Diphtheria (*n* ^1^ = 1224)	<1 year	39	6	3	7	0	0	0	0	4
	1–4 years	297	27	18	202	215	6	0	0	44
	5–9 years	409	21	38	105	198	172	5	1	36
	10–14 years	84	7	4	12	22	49	3	0	11
	15–18 years	20	0	0	1	0	2	3	0	2
	Total	849	61	63	327	435	229	11	1	97
Tetanus (*n* ^1^ = 1229)	<1 year	39	6	3	7	0	0	0	0	4
	1–4 years	295	27	18	204	216	6	0	0	43
	5–9 years	408	24	35	101	201	170	5	1	40
	10–14 years	82	7	4	12	21	50	3	0	13
	15–18 years	20	0	0	1	0	2	3	0	2
	Total	844	64	60	325	438	228	11	1	102
Hepatitis B (*n* ^1^ = 1055)	<1 year	39	8	3	4	1	0	0	0	4
	1–4 years	341	37	33	297	59	2	0	0	40
	5–9 years	511	23	32	339	34	12	0	1	33
	10–14 years	108	3	7	62	4	0	0	0	8
	15–18 years	19	0	0	4	1	0	0	0	4
	Total	1018	71	75	706	99	14	0	1	89

(**^1^**) *n* = total number of children with documentation. (**^2^**) 0 doses = children without documentation. (**^3^**) Missing = children with documentation but no information about the number of doses received.

## Appendix A

**Table A1:** Percentages of seropositive and seronegative children in the study for diphtheria, tetanus, and hepatitis B, by age group.

Age Group	Serologic Status	Diphtheria	Tetanus	Hepatitis B
<1 year	Seropositive	12 (85.7%)	42 (79.2%)	36 (62.1%)
	Seronegative	2 (14.3%)	11 (20.8%)	22 (37.9%)
1–4 years	Seropositive	56 (86.2%)	645 (81.3%)	569 (71.2%)
	Seronegative	9 (13.8%)	148 (18.7%)	230 (28.8%)
5–9 years	Seropositive	73 (90.1%)	799 (82.5%)	541 (55.4%)
	Seronegative	8 (9.9%)	169 (17.5%)	435 (44.6%)
10–14 years	Seropositive	12 (92.3%)	149 (78.0%)	93 (48.7%)
	Seronegative	1 (7.7%)	42 (22.0%)	98 (51.3%)
15–18 years	Seropositive	1 (100.0%)	20 (71.4%)	12 (44.4%)
	Seronegative	0 (0.0%)	8 (28.6%)	15 (55.6%)

**Table A2:** Comparison between immunisation status of IAC reported in the documentation and serology for diphtheria, by continent of origin.

Continent	Immunisation Status According to Documentation	Immunisation Status According to Serology		
		Seropositive	Seronegative	Missing
Africa (*n* = 360)	Recorded	4 (1.1%)	0 (0.0%)	70 (19.4%)
	Not recorded	17 (4.7%)	9 (2.5%)	259 (71.9%)
	Missing	−	−	1 (0.3%)
America (*n* = 449)	Recorded	15 (3.3%)	0 (0.0%)	240 (53.5%)
	Not recorded	19 (4.2%)	3 (0.7%)	171 (38.1%)
	Missing	−	−	1 (0.2%)
Asia (*n* = 420)	Recorded	12 (2.9%)	1 (0.2%)	228 (54.3%)
	Not recorded	25 (6.0%)	4 (1.0%)	150 (35.7%)
	Missing	−	−	0 (0.0%)
Europe (*n* = 844)	Recorded	45 (5.3%)	2 (0.2%)	607 (71.9%)
	Not recorded	17 (2.0%)	1 (0.1%)	172 (20.4%)
	Missing	−	−	0 (0.0%)

**Table A3:** Comparison between immunisation status of IAC reported in the documentation and serology for tetanus, by continent of origin.

Continent	Immunisation Status According to Documentation	Immunisation Status According to Serology		
		Seropositive	Seronegative	Missing
Africa (*n* = 360)	Recorded	60 (16.7%)	14 (3.9%)	3 (0.8%)
	Not recorded	158 (43.9%)	115 (31.9%)	10 (2.8%)
	Missing	−	−	0 (0.0%)
America (*n* = 449)	Recorded	226 (50.3%)	27 (6.0%)	4 (0.9%)
	Not recorded	155 (34.5%)	34 (7.6%)	2 (0.4%)
	Missing	−	−	1 (0.2%)
Asia (*n* = 420)	Recorded	192 (45.7%)	48 (11.4%)	4 (1.0%)
	Not recorded	116 (27.6%)	53 (12.6%)	7 (1.7%)
	Missing	−	−	0 (0.0%)
Europe (*n* = 844)	Recorded	581 (68.8%)	64 (7.6%)	6 (0.7%)
	Not recorded	168 (19.9%)	22 (2.6%)	3 (0.4%)
	Missing	−	−	0 (0.0%)

**Table A4:** Comparison between immunisation status of IAC reported in the documentation and serology for hepatitis B, by continent of origin.

Continent	Immunisation Status According to Documentation	Immunisation Status According to Serology		
		Seropositive	Seronegative	Missing
Africa (*n* = 360)	Recorded	35 (9.7%)	18 (5.0%)	0 (0.0%)
	Not recorded	130 (36.1%)	172 (47.8%)	5 (1.4%)
	Missing	−	−	0 (0.0%)
America (*n* = 449)	Recorded	143 (31.9%)	63 (14.0%)	2 (0.4%)
	Not recorded	150 (33.4%)	90 (20.0%)	1 (0.2%)
	Missing	−	−	0 (0.0%)
Asia (*n* = 420)	Recorded	159 (37.9%)	69 (16.4%)	3 (0.7%)
	Not recorded	111 (26.4%)	77 (18.3%)	1 (0.2%)
	Missing	−	−	0 (0.0%)
Europe (*n* = 844)	Recorded	395 (46.8%)	158 (18.7%)	7 (0.8%)
	Not recorded	129 (15.3%)	153 (18.1%)	2 (0.2%)
	Missing	−	−	0 (0.0%)

**Table A5:** Comparison between immunisation status of IAC reported in the documentation and serology for diphtheria, by age group.

Age Group	Immunisation Status According to Documentation	Immunisation Status According to Serology		
		Seropositive	Seronegative	Missing
<1 year (*n* = 59)	Recorded	2 (3.4%)	0 (0.0%)	18 (30.5%)
	Not recorded	10 (17.0%)	2 (3.4%)	27 (45.8%)
	Missing	−	−	0 (0.0%)
1–4 years (*n* = 809)	Recorded	27 (3.3%)	2 (0.3%)	483 (59.7%)
	Not recorded	29 (3.6%)	7 (0.9%)	261 (32.3%)
	Missing	−	−	0 (0.0%)
5–9 years (*n* = 985)	Recorded	39 (4.0%)	1 (0.1%)	536 (54.4%)
	Not recorded	34 (3.5%)	7 (0.7%)	366 (37.2%)
	Missing	−	−	2 (0.2%)
10–14 years (*n* = 192)	Recorded	7 (3.7%)	0 (0.0%)	101 (52.6%)
	Not recorded	5 (2.6%)	1 (0.5%)	78 (40.6%)
	Missing	−	−	0 (0.0%)
15–18 years (*n* = 28)	Recorded	1 (3.6%)	0 (0.0%)	7 (25.0%)
	Not recorded	0 (0.0%)	0 (0.0%)	20 (71.4%)
	Missing	−	−	0 (0.0%)

**Table A6:** Comparison between immunisation status of IAC reported in the documentation and serology for tetanus, by age group.

Age Group	Immunisation Status According to Documentation	Immunisation Status According to Serology		
		Seropositive	Seronegative	Missing
<1 year (*n* = 59)	Recorded	18 (30.5%)	1 (1.7%)	1 (1.7%)
	Not recorded	24 (40.7%)	10 (17.0%)	5 (8.5%)
	Missing	−	−	0 (0.0%)
1–4 years (*n* = 809)	Recorded	436 (53.9%)	72 (8.9%)	6 (0.7%)
	Not recorded	209 (25.8%)	76 (9.4%)	10 (1.2%)
	Missing	−	−	0 (0.0%)
5–9 years (*n* = 985)	Recorded	513 (52.1%)	55 (5.6%)	9 (0.9%)
	Not recorded	287 (29.1%)	113 (11.5%)	7 (0.7%)
	Missing	−	−	1 (0.1%)
10–14 years (*n* = 192)	Recorded	85 (44.3%)	24 (12.5%)	1 (0.5%)
	Not recorded	64 (33.3%)	18 (9.4%)	0 (0.0%)
	Missing	−	−	0 (0.0%)
15–18 years (*n* = 28)	Recorded	7 (25.0%)	1 (3.6%)	0 (0.0%)
	Not recorded	13 (46.4%)	7 (25.0%)	0 (0.0%)
	Missing	−	−	0 (0.0%)

**Table A7:** Comparison between immunisation status of IAC reported in the documentation and serology for hepatitis B, by age group.

Age Group	Immunisation Status According to Documentation	Immunisation Status According to Serology		
		Seropositive	Seronegative	Missing
<1 year (*n* = 59)	Recorded	17 (28.8%)	3 (5.1%)	0 (0.0%)
	Not recorded	19 (32.2%)	19 (32.2%)	1 (1.7%)
	Missing	−	−	0 (0.0%)
1–4 years (*n* = 809)	Recorded	359 (44.4%)	103 (12.7%)	6 (0.7%)
	Not recorded	210 (26.0%)	127 (15.7%)	4 (0.5%)
	Missing	−	−	0 (0.0%)
5–9 years (*n* = 985)	Recorded	305 (31.0%)	164 (16.7%)	5 (0.5%)
	Not recorded	236 (24.0%)	271 (27.5%)	4 (0.4%)
	Missing	−	−	0 (0.0%)
10–14 years (*n* = 192)	Recorded	48 (25.0%)	35 (18.2%)	1 (0.5%)
	Not recorded	45 (23.4%)	63 (32.8%)	0 (0.0%)
	Missing	−	−	0 (0.0%)
15–18 years (*n* = 28)	Recorded	3 (10.7%)	3 (10.7%)	0 (0.0%)
	Not recorded	10 (35.7%)	12 (42.9%)	0 (0.0%)
	Missing	−	−	0 (0.0%)

**Table A8:** Univariate and multivariate analyses for factors associated with seronegativity for tetanus.

**Possible Risk Factors**		**Seronegative Children/Total**	**Univariate Analysis**			**Multivariate Analysis**		
			**Odds Ratio**	***p***	**95% CI**	**Odds Ratio**	***p***	**95% CI**
**Age group**	0–4 years	159/846 (18.79%)	1					
	5–9 years	169/968 (17.46%)	0.914	0.461	0.72–1.16			
	10–18 years	50/219 (22.83%)	1.278	0.181	0.89–1.83			
**Continent of origin**	Europe	86/835 (10.30%)	1			1		
	America	61/442 (13.80%)	1.394	0.063	0.98–1.98	1.185	0.355	0.83–1.70
	Asia	101/409 (24.69%)	2.856	<0.001	2.08–3.92	2.419	<0.001	1.75–3.35
	Africa	130/347 (37.46%)	5.218	<0.001	3.82–7.12	3.605	<0.001	2.57–5.07
**Immunisation status according to documentation**	Not Recorded	224/820 (27.32%)	1			1		
	Recorded	153/1212 (12.62%)	0.384	<0.001	0.31–<0.48	0.504	<0.001	0.39–0.65
**Sex**	Males	221/1201 (18.40%)	1					
	Females	157/832 (18.87%)	1.031	0.789	0.82–1.29			
**Period of arrival in Italy**	Before 2009	2/50 (4.00%)	0.187	0.022	0.04–0.79	0.176	0.019	0.04–0.75
	2009–2011	78/429 (18.18%)	1			1		
	2012–2014	113/691 (16.35%)	0.880	0.429	0.64–1.21	0.963	0.824	0.69–1.34
	2015–2019	170/784 (21.68%)	1.246	0.149	0.92–1.68	1.428	0.028	1.04–1.96
	Missing	15/79 (18.99%)	1.055	0.865	0.57–1.95	1.043	0.898	0.55–1.97

**Table A9:** Univariate and multivariate analyses for factors associated seronegativity for diphtheria.

**Possible Risk Factors**		**Seronegative Children/Total**	**Univariate Analysis**			**Multivariate Analysis**		
			**Odds Ratio**	***p***	**95% CI**	**Odds Ratio**	***p***	**95% CI**
**Age group**	0–4 years	11/66 (16.67%)	1					
	5–9 years	8/71 (11.27%)	0.635	0.364	0.24–1.69			
	10–18 years	1/12 (8.33%)	0.455	0.472	0.05–3.89			
**Continent of origin**	Europe	3/56 (5.36%)	1			1		
	America	3/34 (8.82%)	1.710	0.527	0.32–9.00	1.150	0.873	0.21–6.38
	Asia	5/34 (14.71%)	3.046	0.146	0.68–13.67	2.084	0.355	0.44–9.87
	Africa	9/25 (36%)	9.937	0.002	2.40–41.16	5.553	0.026	1.23–25.05
**Immunisation status according to documentation**	Not Recorded	17/80 (21.25%)	1			1		
	Recorded	3/69 (4.35%)	0.316	0.049	0.10–1.00	0.258	0.05	0.07–1.00
**Sex**	Males	10/93 (10.75%)	1					
	Females	10/56 (17.86%)	1.804	0.222	0.70–4.65			
**Period of arrival in Italy**	Before 2009	1/31 (3.23%)	0.182	0.106	0.2–1.44			
	2009–2011	15/97 (15.46%)	1					
	2012–2014	1/2 (50.00%)	5.467	0.239	0.32–92.25			
	2015–2019	2/5 (40.00%)	3.644	0.176	0.56–23.69			
	Missing	1/14 (7.14%)	0.421	0.420	0.05–3.46			

**Table A10:** Univariate and multivariate analyses for factors associated with seronegativity for hepatitis B.

**Possible Risk Factors**		**Seronegative Children/Total**	**Univariate Analysis**			**Multivariate Analysis**		
			**Odds Ratio**	***p***	**95% CI**	**Odds Ratio**	***p***	**95% CI**
**Age group**	0–4 years	252/857 (29.40%)	1			1		
	5–9 years	435/976 (44.57%)	1.930	<0.001	1.59–2.34	2.086	<0.001	1.69–2.57
	10–18 years	113/219 (51.60%)	2.559	<0.001	1.89–3.47	2.686	<0.001	1.94–3.72
**Continent of origin**	Europe	311/835 (37.25%)	1			1		
	America	153/446 (34.30%)	0.880	0.297	0.69–1.12	0.627	<0.001	0.48–0.81
	Asia	146/416 (35.10%)	0.911	0.457	0.71–1.16	0.869	0.292	0.67–1.13
	Africa	190/355 (53.52%)	1.94	<0.001	1.51–2.49	1.597	0.001	1.20–2.13
**Immunisation status according to documentation**	Not Recorded	492/1010 (48.71%)	1			1		
	Recorded	308/1040 (29.62%)	0.443	<0.001	0.37–0.53	0.485	<0.001	0.40–0.59
**Sex**	Males	492/1218 (40.39%)	1					
	Females	308/834 (36.93%)	0.864	0.114	0.72–1.04			
**Period of arrival in Italy**	Before 2009	13/49 (26.53%)	0.568	0.095	0.29–1.10	0.495	0.046	0.25–0.99
	2009–2011	169/435 (38.85%)	1			1		
	2012–2014	231/695 (33.24%)	0.784	0.055	0.61–1.01	0.786	0.071	0.60–1.02
	2015–2019	360/791 (45.51%)	1.315	0.024	1.04–1.67	1.440	0.005	1.12–1.86
	Missing	27/82 (32.93%)	0.635	0.311	0.47–1.27	0.769	0.322	0.46–1.29
